# Boundary-aware geometric Mamba network for breast tumor segmentation in 3D MRI

**DOI:** 10.3389/fonc.2026.1972153

**Published:** 2026-10-09

**Authors:** Zhejun Kuang, Yuxuan Zhu, Dawen Sun, Jian Zhao

**Affiliations:** 1 College of Computer Science and Technology, Changchun University, Changchun, Jilin, China; 2 Jilin Provincial Key Laboratory of Human Health Status Identification Function & Enhancement, Changchun, Jilin, China; 3 Key Laboratory of Intelligent Rehabilitation and Barrier-Free for the Disabled, Changchun University, Ministry of Education, Changchun, Jilin, China

**Keywords:** boundary-aware segmentation, breast tumor segmentation, geometric transformation, magnetic resonance imaging, Mamba, medical image segmentation, state-space model, structural representation learning

## Abstract

**Introduction:**

Accurate breast tumor segmentation in Dynamic Contrast-Enhanced Magnetic Resonance Imaging (DCE-MRI) is important for reliable diagnosis and treatment planning. However, complex tumor morphology, diverse spatial structures, and indistinct boundaries remain challenging for existing segmentation methods. Convolutional neural network-based methods have limited ability to model long-range dependencies, while Transformer-based methods often introduce substantial computational overhead. Although Mamba-based approaches provide an efficient alternative, conventional scanning strategies may suffer from directional bias and insufficiently capture three-dimensional structural variations.

**Methods:**

We propose a Boundary-Aware Geometric Mamba Network (BAGMNet) for three-dimensional breast tumor segmentation. BAGMNet establishes a structural representation learning framework that combines geometric modeling, multi-directional state-space propagation, and boundary-aware feature refinement. The Geometric Mamba Encoder constructs complementary spatial representations through multiple geometric transformations, while the Multi-directional Selective State Space module enhances three-dimensional dependency modeling and alleviates the directional bias of conventional Mamba scanning. The Gated State-space Fusion and Adaptive Skip Recalibration modules further integrate local structural details with global contextual information and enhance boundary-related representations during feature propagation.

**Results:**

Extensive experiments were conducted on the ISPY1 and Yunnan breast MRI datasets. BAGMNet achieved Dice scores of 70.34% and 79.14%, with average surface distances of 9.79 mm and 8.96 mm on the ISPY1 and Yunnan datasets, respectively. Compared with representative convolutional neural network-, Transformer-, and Mamba-based methods, BAGMNet achieved competitive performance in both regional segmentation accuracy and boundary localization.

## 1 Introduction

Breast cancer is one of the most prevalent malignant diseases among women worldwide (1), and early diagnosis together with precise treatment is essential for improving patient survival (2). Owing to its superior soft-tissue contrast and multi-parametric imaging capability, Magnetic Resonance Imaging (MRI) has been widely adopted for breast tumor detection, treatment response assessment, and preoperative planning (3, 4). Accurate breast tumor segmentation is important for lesion localization, tumor volume estimation, radiotherapy target delineation, and subsequent disease progression and radiomics analyses (5). In practice, manual tumor delineation is time-consuming and relies heavily on clinical expertise. Moreover, variations between observers may result in inconsistent tumor boundaries (4). Therefore, developing an accurate, automatic, and robust breast MRI tumor segmentation method remains an important research topic (6).

Despite the remarkable success of deep learning in medical image segmentation, automatic breast tumor segmentation from MRI remains highly challenging (6). As illustrated in **Figure 1**, breast tumors usually exhibit substantial structural heterogeneity, with significant variations in size, shape, internal texture, and enhancement patterns across different patients (7, 8). Even within a single lesion, tumor morphology is often highly irregular and spatially inconsistent (9). Moreover, infiltrative tumor growth, low tissue contrast, and MRI imaging noise jointly result in ambiguous, discontinuous, or partially missing tumor boundaries, making segmentation models prone to boundary displacement, contour discontinuity, and regional omission (10, 11). Therefore, accurately perceiving complex tumor structures while recovering ambiguous boundaries remains one of the major challenges in breast MRI segmentation (8, 9).

**Figure 1:**
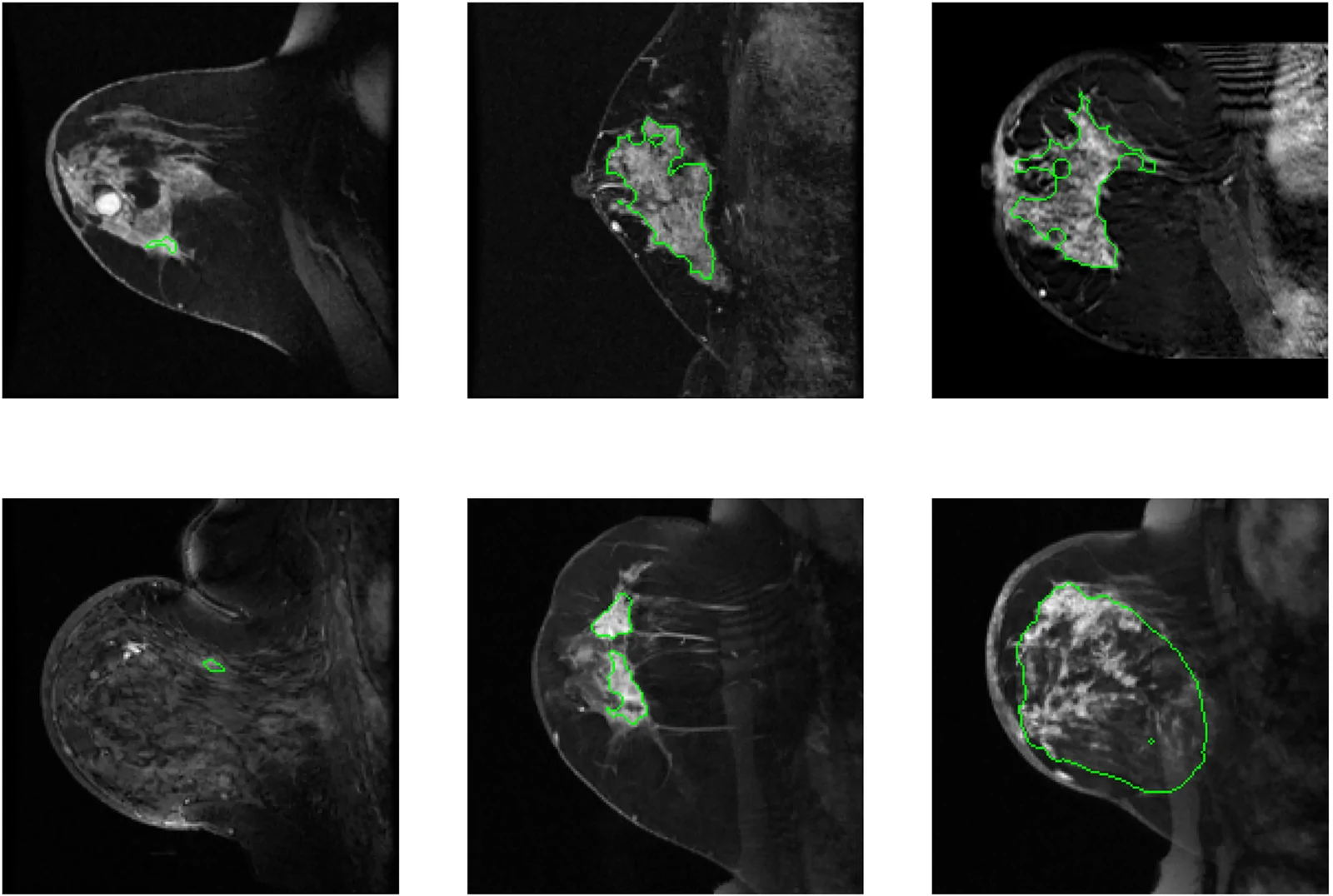
Representative examples of breast tumors with diverse sizes, shapes, boundary complexity, and internal heterogeneity in MRI. Green contours indicate tumor regions (ISPY1).

Besides lesion complexity, breast MRI data are commonly collected from multiple clinical centers with different imaging devices, acquisition protocols, and contrast-enhancement strategies, resulting in considerable domain shifts across datasets (12). Such variation across imaging centers makes it more difficult for a model to learn structural features that remain stable across cases. For breast tumor segmentation, the model needs to preserve fine anatomical details while also capturing spatial relationships over a broader range. This becomes particularly important near poorly defined tumor boundaries, where local appearance alone may not provide sufficient information (13).

U-Net and its variants have been widely used as the basis for medical image segmentation because of their ability to combine features from different spatial scales (14). CNN-based implementations of this encoder–decoder design further extract hierarchical features through successive convolutional operations and have achieved strong performance across a range of medical segmentation tasks. However, CNN-based methods mainly rely on local convolution operations and are inherently limited by restricted receptive fields, which makes them insufficient for capturing long-range spatial dependencies in three-dimensional MRI volumes (15). Consequently, when dealing with highly heterogeneous breast tumors with complex morphological variations, CNN-based approaches may suffer from incomplete structural perception and inaccurate boundary delineation.

To enhance global contextual modeling, Transformer-based methods introduce self-attention mechanisms to establish long-range dependencies between distant regions (16, 17). Compared with CNNs, these approaches provide stronger global representation capability and have demonstrated promising performance in medical image segmentation. Nevertheless, the self-attention mechanism requires computational complexity that grows quadratically with the number of input tokens, resulting in substantial computational and memory overhead for high-resolution three-dimensional medical images. Moreover, the global interaction strategy of Transformers does not explicitly emphasize local structural continuity, which may limit their ability to accurately preserve fine-grained boundary details in ambiguous tumor regions.

Recently, State-Space Models (SSMs), particularly Mamba, have emerged as an efficient alternative for long-range dependency modeling due to their linear computational complexity and selective state propagation mechanism (18). Compared with Transformer-based architectures, Mamba-based methods achieve more efficient global feature modeling and have shown great potential in volumetric medical image segmentation (19, 20). However, existing Mamba-based approaches generally perform feature propagation following predefined scanning strategies. Such sequential modeling mechanisms introduce directional bias (anisotropy), limiting the comprehensive perception of complex three-dimensional tumor structures from multiple spatial orientations. Although several multi-directional scanning variants have been proposed to mitigate this anisotropy, they generally traverse the same spatial arrangement along different directions, which only changes the propagation order rather than the underlying voxel adjacency; consequently, the structural dependencies established along different scans largely overlap and remain constrained by the fixed spatial organization of the input volume. Furthermore, current Mamba-based methods primarily focus on enhancing global dependency modeling, while the preservation of local structural details around ambiguous boundaries remains insufficient, potentially leading to over-smoothed contours and incomplete recovery of fine-grained anatomical structures (15).

Therefore, although CNN-, Transformer-, and Mamba-based methods have significantly advanced medical image segmentation, effectively balancing global structural perception, multi-directional spatial modeling, and precise boundary preservation remains a critical challenge for heterogeneous breast tumor segmentation.

Further analysis suggests that the above limitations are closely related to how spatial and structural information is modeled. Most existing methods rely on a single spatial view when learning tumor representations, which may not be sufficient to describe the morphological variations of tumors across different spatial arrangements. In addition, although Mamba provides an efficient mechanism for modeling long-range dependencies, its fixed scanning strategy tends to favor specific directions and may result in anisotropic feature representations in 3D space. The feature fusion process also remains an important issue. Conventional fusion and skip-connection schemes mainly combine features from different network levels without explicitly considering their structural relevance. As a result, fine boundary information may be weakened during feature propagation, while irrelevant background responses can also be passed to the decoder.

These observations motivate the design of a Boundary-Aware Geometric Mamba Network (BAGMNet) for breast tumor MRI segmentation. The network is built around a structural representation learning strategy that considers tumor morphology from multiple spatial views and preserves boundary information during feature decoding.

The encoder of BAGMNet adopts a Geometric Mamba Encoder (GME), where geometric transformations are applied to construct complementary views of the input features. Unlike multi-directional scanning strategies that traverse the same arrangement along different directions, GME explicitly reorganizes the spatial layout so that the resulting serialization paths operate over different voxel neighborhoods and thus establish genuinely different structural dependencies. These views provide additional spatial arrangements for subsequent state-space modeling and allow the encoder to describe tumor morphology beyond a single spatial organization. Within GME, the Multi-directional Selective State Space (MSS3D) module propagates state information along each reorganized trajectory and further regularizes directional structural continuity along the orthogonal anatomical axes. This design reduces the dependence on a fixed scanning direction and provides a more balanced representation of three-dimensional structures.

Structural information is further considered during feature decoding. The Gated State-space Fusion (GSF) module combines local convolutional features with long-range state-space features, with a learnable gate controlling their relative contributions. The Adaptive Skip Recalibration (ASR) module operates on skip features to retain informative structural responses and suppress less relevant background information. Together, these designs allow BAGMNet to maintain structural details from the encoder while refining the representation of ambiguous tumor boundaries in the decoder.

The main contributions of this work can be summarized as follows:

- We propose a Boundary-Aware Geometric Mamba Network (BAGMNet) for breast tumor segmentation in MRI. The network is designed around a unified structural representation learning framework that considers geometric variation, directional state propagation, and boundary-aware feature decoding.
- We develop a Geometric Mamba Encoder (GME) with the Multi-directional Selective State Space (MSS3D) module. GME constructs complementary spatial views through geometric transformations, while MSS3D performs state propagation along multiple directions to reduce the directional bias associated with fixed Mamba scanning.
- We develop a Gated State-space Fusion (GSF) module and an Adaptive Skip Recalibration (ASR) module for structural feature decoding. GSF controls the interaction between local and long-range features, while ASR selectively recalibrates skip features to preserve informative boundary responses and suppress redundant background information.
- We evaluate BAGMNet on the ISPY1 and Yunnan breast MRI datasets. The results show that the proposed method maintains competitive segmentation performance across both datasets and performs favorably against representative CNN-, Transformer-, and Mamba-based approaches.

## 2 Related work

### 2.1 CNN-based methods

#### 2.1.1 Encoder–decoder architectures

Convolutional Neural Networks (CNNs) have long dominated medical image segmentation owing to their strong capability for local feature extraction. Among them, U-Net has become one of the most influential encoder–decoder architectures by employing a symmetric downsampling–upsampling framework together with skip connections to effectively integrate low-level spatial details and high-level semantic information (21). Following U-Net, a variety of CNN-based architectures have been proposed to improve feature representation and segmentation accuracy. For example, V-Net extended U-Net to volumetric medical image segmentation by adopting three-dimensional convolutions to model volumetric contextual information (22). UNet++ introduced nested dense skip pathways to narrow the semantic gap between encoder and decoder features, allowing information from different network depths to be combined more effectively (14). nnU-Net took a different approach by introducing a self-configuring training framework, which automatically determines preprocessing procedures, network configurations, and optimization settings according to the characteristics of each segmentation task (23).

Residual and dense connectivity have also been incorporated into encoder–decoder architectures to improve information flow across network stages. ResUNet and DenseUNet are representative examples, using residual and dense connections to facilitate gradient propagation and feature reuse (24, 25). Despite these improvements, the feature interactions in such CNN-based architectures are still primarily performed through local convolutional operations. Their limited receptive fields therefore make it difficult to establish long-range contextual relationships, especially in volumetric medical images where relevant structural information can span multiple slices (26).

#### 2.1.2 Multi-scale and attention-enhanced CNNs

CNN-based segmentation methods have also been extended with multi-scale feature extraction and attention mechanisms to better handle the varying size and complex morphology of medical lesions. Multi-scale strategies, such as dilated convolution, pyramid pooling, and multi-resolution feature aggregation, enlarge the effective receptive field and allow features to be collected at different spatial scales. For example, CE-Net combines context encoding with atrous convolution to obtain richer contextual features (26), while DeepLabv3+ uses atrous spatial pyramid pooling to combine semantic information from multiple scales, improving its ability to segment complex anatomical structures (27).

Despite their ability to improve feature selection, these attention mechanisms are generally applied on top of convolutional feature extraction and therefore remain dependent on the receptive field of the underlying CNN. Despite these improvements, the underlying feature interaction in these methods is still governed mainly by convolutional receptive fields. Consequently, their ability to model long-range spatial dependencies remains limited, especially for heterogeneous breast tumors whose structural information can extend across a large three-dimensional region.

#### 2.1.3 3D CNNs and boundary-aware segmentation

For volumetric medical images, 3D CNNs have been widely used for tumor and organ segmentation. Methods such as 3D U-Net and V-Net employ three-dimensional convolutions to directly extract volumetric features, allowing the models to make use of contextual information across adjacent slices and preserve anatomical continuity (22). More recent architectures, including nnFormer and other hybrid CNN-based models, have further explored hierarchical feature learning to improve the representation of volumetric structures (28).

Boundary information is another important consideration in medical image segmentation, particularly when tumors have low contrast with surrounding tissues. Several approaches have therefore introduced additional boundary supervision or explicitly modeled edge and contour information through auxiliary branches, contour constraints, or boundary-aware loss functions. BMFNet, for example, incorporates boundary-aware multi-scale fusion for breast tumor segmentation (10), while shape- and boundary-aware multi-branch networks have been developed to strengthen the representation of discriminative contour features (8). Although these mechanisms help refine ambiguous regions, the underlying feature interaction is still largely based on local convolution. Modeling structural relationships over a larger spatial range while preserving fine boundary information therefore remains difficult. This limitation is especially important in breast tumor MRI, where considerable morphological variation and poorly defined tumor margins may result in fragmented structures, incomplete delineation, and unstable performance across cases (6).

### 2.2 Transformer-based methods

Transformer architectures have been adopted in medical image segmentation to model spatial dependencies beyond the local receptive field of convolution. Their self-attention mechanism relates features from different spatial positions, allowing contextual information to be exchanged across a wider region. TransUNet is a representative hybrid architecture that incorporates a Transformer encoder into the U-Net framework. The encoder extracts global semantic representations, while the CNN-based decoder recovers spatial details from the encoded features, leading to strong results on a range of medical image segmentation tasks (16). Subsequently, Swin UNETR pioneered a hierarchical Transformer encoder for volumetric medical image segmentation and incorporated multi-scale skip connections to facilitate volumetric contextual modeling, further improving segmentation performance for anatomically complex structures (17).

To improve the efficiency of Transformer architectures in high-resolution medical imaging scenarios, hierarchical vision Transformer frameworks have also been proposed. For example, Swin UNETR employs shifted window attention mechanisms to reduce computational complexity through localized attention computation while leveraging hierarchical feature representations to enhance multi-scale structural perception (17). In addition, MISSFormer introduces a multi-scale Transformer encoding strategy to better capture morphological variations of complex lesions (29), whereas nnFormer designs a hybrid local–global attention mechanism specifically for volumetric medical image segmentation, demonstrating strong capability in modeling cross-slice spatial dependencies (28).

Despite their superior performance in global contextual modeling, Transformer-based methods commonly suffer from the quadratic computational complexity of self-attention with respect to input size, leading to considerable memory consumption and computational overhead, particularly in high-resolution 3D MRI scenarios. Moreover, existing methods often place greater emphasis on global semantic modeling while paying insufficient attention to local texture details and fine-grained boundary structures. As a result, boundary over-smoothing and inadequate structural recovery may occur in regions with ambiguous boundaries or low tissue contrast. Particularly in breast tumor MRI segmentation, where lesions exhibit pronounced heterogeneity and complex boundary morphology, relying solely on Transformer-based global modeling remains insufficient for simultaneously capturing long-range dependencies and preserving fine-grained boundary information (6).

### 2.3 State-space models and the Mamba architecture

Recently, State-Space Models (SSMs) have attracted increasing attention due to their ability to achieve efficient long-range dependency modeling with linear computational complexity. Among them, the Mamba architecture employs a Selective State Space Mechanism to dynamically model long-range contextual information and has demonstrated strong performance in vision understanding and sequential modeling tasks (18). Compared with Transformers, Mamba propagates global semantic information more efficiently and therefore shows considerable potential for high-resolution volumetric medical image analysis.

With the rapid development of Mamba-based vision architectures, increasing efforts have been devoted to applying state-space modeling to medical image segmentation. For example, VMamba extends state-space modeling to vision tasks and improves spatial dependency learning through multi-directional scanning strategies (30). U-Mamba integrates Mamba into the U-Net encoder–decoder framework, enhancing global contextual representation while preserving local structural recovery capability (15). SegMamba further exploits state-space mechanisms to model cross-slice continuity in volumetric medical images, thereby improving contextual understanding in three-dimensional segmentation tasks (19). In addition, methods such as Mamba-UNet and MedMamba attempt to combine state-space modeling with convolutional structures to simultaneously exploit local texture information and global contextual dependencies.

Despite their computational efficiency and ability to model long-range dependencies, current Mamba-based segmentation methods still face several challenges. A common design is to propagate states along a predefined scanning direction or within a fixed spatial arrangement. Such a strategy does not explicitly account for structural changes across different orientations and may introduce directional bias when dealing with tumors with irregular and heterogeneous morphology.

Several works have attempted to alleviate this directional anisotropy through multi-directional or cross-directional scanning. For example, VMamba adopts a cross-scan that traverses the same feature map along four directions, and similar strategies have been extended to volumetric data by adding reverse and orthogonal traversals. Although these approaches increase the diversity of propagation directions, they still operate on a fixed spatial arrangement: reversing or partitioning the scanning order changes the traversal direction but does not alter the voxel adjacency induced by the spatial organization. As a result, the structural dependencies established along different scans largely overlap, and the anisotropy introduced by sequential state propagation is only partially compensated. Moreover, simply processing the same set of features along multiple directions tends to aggregate redundant information, and the gain obtained by increasing the number of scanning directions rapidly saturates without fundamentally expanding the structural relationships that can be captured. These observations indicate that mitigating directional bias requires going beyond varying the traversal direction over a single spatial arrangement.

Global contextual modeling alone, however, is not sufficient for precise boundary reconstruction. Fine-grained textures and small changes along the tumor contour can be lost during feature extraction, making the predicted boundaries overly smooth or incomplete. This problem becomes more pronounced in regions with weak tissue contrast, where the distinction between the tumor and surrounding tissue is less evident. Feature fusion in encoder–decoder architectures is another concern. Direct concatenation or element-wise addition is still widely used for combining features from different levels, but these operations provide limited control over which information should be retained or suppressed around complex tumor boundaries.

These observations highlight different limitations of the existing segmentation paradigms. CNNs provide effective local feature extraction but have difficulty modeling distant spatial relationships. Transformers offer stronger global interaction through self-attention, at the cost of considerably higher computational requirements for volumetric data. Mamba provides a more efficient alternative for long-range modeling, yet its existing formulations do not fully account for directional variations, local boundary structures, and the selective fusion of multi-level features. Based on these considerations, we propose the Boundary-Aware Geometric Mamba Network (BAGMNet) for breast tumor MRI segmentation. The network combines multi-directional geometric modeling, local–global feature interaction, and boundary-aware feature recalibration to better represent heterogeneous tumor structures and preserve ambiguous boundaries.

## 3 Methods

### 3.1 Architecture overview

Breast tumors in 3D magnetic resonance images (MRI) usually exhibit substantial structural heterogeneity, with significant variations in morphology, size, growth orientation, and boundary appearance across different patients. Meanwhile, the low contrast between tumor tissues and surrounding normal structures often leads to ambiguous or even discontinuous boundaries, making it difficult to establish discriminative structural representations. Existing 3D medical image segmentation methods generally perform feature learning under fixed spatial arrangements, limiting their ability to perceive structural variations from different spatial directions and explicitly model boundary transitions.

To address these challenges, we propose a structural representation learning framework for 3D breast tumor segmentation, as illustrated in **Figure 2**. The proposed network follows a U-shaped encoder–decoder architecture, consisting of a Geometric Mamba Encoder (GME), a Gated State-space Fusion (GSF) block, an Adaptive Skip Recalibration (ASR) block, and a lightweight decoder. Through spatial reorganization, state-space modeling, adaptive structural integration, and cross-level structural preservation, the proposed framework progressively establishes increasingly discriminative structural representations from local anatomical details to global structural semantics.

**Figure 2:**
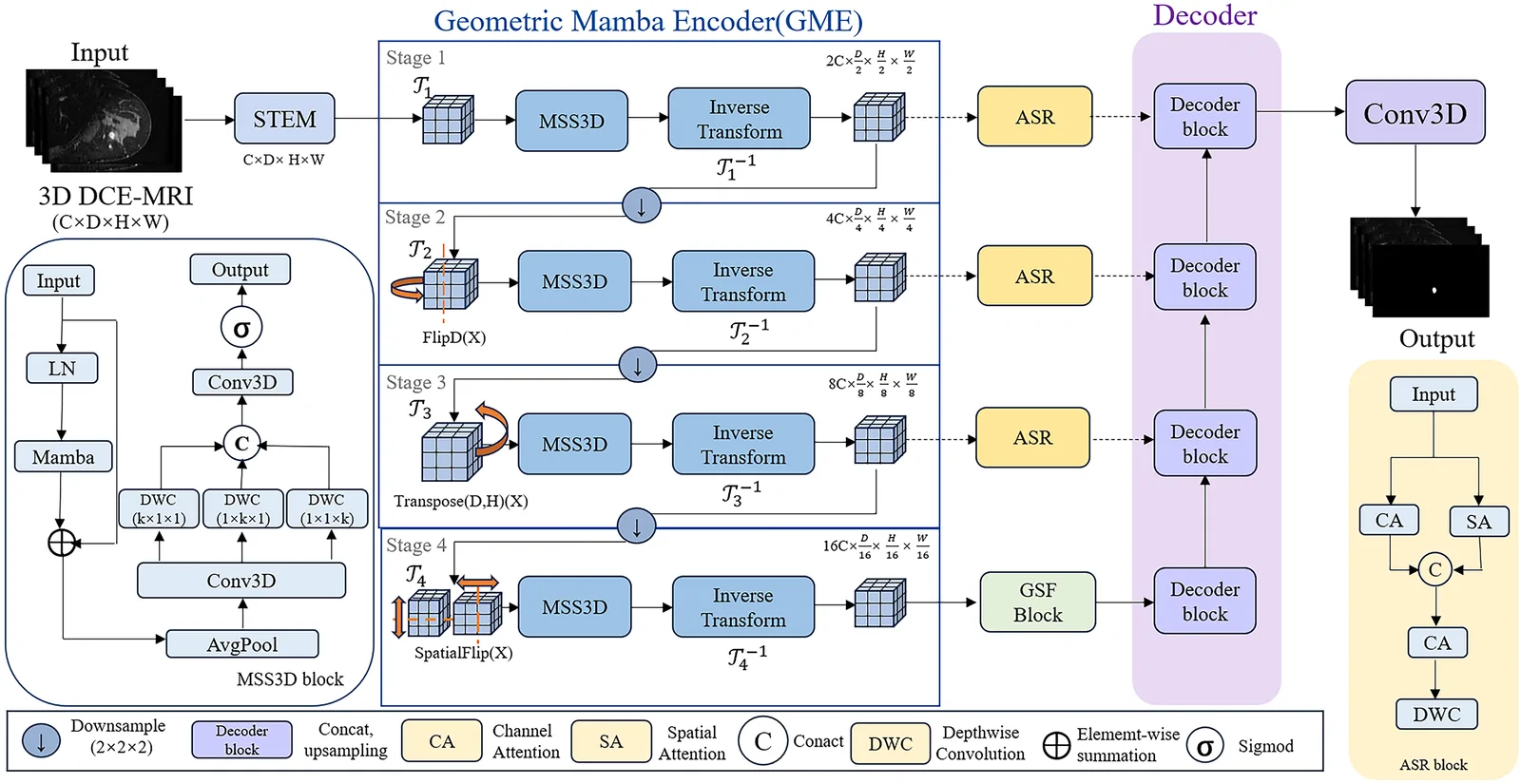
BAGMNet consists of a Geometric Mamba Encoder (GME), a Gated State-space Fusion module (GSF), and an Adaptive Skip Recalibration module (ASR) within an encoder–decoder framework.

Specifically, the GME block first constructs complementary spatial representations through geometric transformations and subsequently performs multi-directional state propagation using the proposed Multi-directional Selective State Space (MSS3D) module, thereby enhancing long-range structural connectivity under diverse spatial arrangements. The GSF block then adaptively integrates complementary local and global structural representations. Furthermore, the proposed Boundary-Aware Modulation (BAM) module constructs a boundary-aware structural prior in the learned structural embedding space, which guides the Dynamic Channel Attention (DCA) module to perform structure-guided structural interaction. Finally, the ASR block recalibrates encoder features before cross-level transmission, preserving structurally informative representations for subsequent decoding. The successive refinement of structural features improves segmentation in heterogeneous breast tumors, particularly around poorly defined boundaries.

### 3.2 Geometric Mamba Encoder block

In 3D magnetic resonance imaging (MRI), breast tumors can vary considerably in morphology, growth direction, and boundary continuity, making their structural appearance highly heterogeneous. Existing 3D segmentation networks generally perform feature learning under a fixed spatial organization, where voxels are serialized following a predefined arrangement throughout the encoding process. Although such a strategy is effective for semantic representation learning, the structural dependencies established by sequential modeling are inherently constrained by the underlying spatial organization, limiting the diversity of structural relationships that can be captured for highly heterogeneous tumors.

To overcome this limitation, we propose the Geometric Mamba Encoder (GME) block, which progressively constructs complementary structural representations through spatial reorganization and long-range dependency modeling. Instead of directly modifying the state-space model itself, GME first reorganizes the spatial arrangement of the input feature volume before dependency modeling. Since different spatial organizations naturally induce different serialization paths, complementary state propagation trajectories can be established while preserving the original anatomical information. Consequently, the network is able to perceive heterogeneous tumor structures from multiple structural dependency perspectives rather than relying on a single fixed propagation path.

It is important to distinguish the proposed geometric reorganization from multi-directional scanning strategies that process the same set of features along different traversal directions. In the latter case, the spatial organization of the input volume remains unchanged, so the voxel adjacency induced by serialization is preserved across scans; reversing or partitioning the traversal order only changes the propagation direction, and the structural dependencies established along different scans therefore largely overlap. In contrast, the operators in Ω explicitly reorganize the spatial layout of the input volume: flipping and transposition alter which voxels become adjacent in the serialized sequence, so each reorganized view corresponds to a genuinely different set of local neighborhoods and long-range dependencies. In particular, the depth–height transposition exchanges two anatomical axes, enabling state propagation to establish cross-axis structural relationships that cannot be obtained by simply traversing the original arrangement along additional directions. After dependency modeling, the inverse transformations restore all features to the original coordinate system, and the complementary dependencies established under different spatial organizations are aggregated in the same anatomical reference rather than being averaged over redundant traversals of a single arrangement. Consequently, the gain of GME originates from expanding the structural relationships that can be captured, rather than from repeating the processing of the same features in different orientations.

As illustrated in **Figure 2**, the proposed GME block consists of three successive stages: Spatial Reorganization, Multi-directional Selective State Space (MSS3D) Modeling, and Feature Reconstruction. Specifically, let $X_{l-1}\in {\mathbb{R}}^{C_{1-1}\times D_{l-1}\times H_{l-1}\times W_{l-1}}$ denote the input feature of the l-th encoding stage ( l=1,…,4), with X0 being the STEM output. At each stage, the feature volume is reorganized by a single stage-specific geometric operator, as defined in Equation 1.

(1) $${\tilde{X}}_{l}=T_{l}(X_{l-1}),$$

where the operators are assigned to the four encoding stages in a fixed order, as listed in Equation 2.

(2) $$\left(T_{1},T_{2},T_{3},\;T_{4}\right)=\left(ℐ,{\text{Flip}}_{D},{\text{Transpose}}_{\left(D,\;H\right)},\text{SpatialFlip}\right),$$

corresponding to the identity mapping, depth flipping, depth–height transposition, and spatial flipping, respectively. Rather than applying all transformations in parallel within a single stage, each stage exposes the state-space model to one complementary spatial organization, and the four organizations are accumulated progressively across the encoder. These operations preserve the anatomical content while constructing complementary serialization paths for subsequent dependency modeling.

The reorganized feature volume is subsequently processed by the proposed MSS3D module to establish long-range structural dependencies under the corresponding serialization path. After dependency modeling, the transformed feature is restored to the original spatial coordinate system through the paired inverse transformation, and the 2×2×2 downsampling is then applied. The overall process of the *l*-th GME stage is formulated in Equation 3 as

(3) $$X_{l}=D\left(T_{l}^{-1}\left(F_{\text{MSS}3\text{D}}\left({\tilde{X}}_{l}\right)\right)\right)=D\left(T_{l}^{-1}\left(F_{\text{MSS}3\text{D}}\left(T_{l}\left(X_{l-1}\right)\right)\right)\right),$$

where *F*_MSS3D_(·) denotes the proposed Multi-directional Selective State Space modeling, $T_{l}^{-1}(\cdot )$ is the inverse of the stage-specific reorganization that restores the feature to the original coordinate system ( ℐ, FlipD, and SpatialFlip are self-inverse, while the inverse of ${\text{Transpose}}_{\left(\text{D,H}\right)}$ is ${\text{Transpose}}_{\left(\text{H,D}\right)}$), *D*(·) denotes the 2×2×2 downsampling operation, and *X_l_* is the output feature of the *l*-th GME stage.

By coupling stage-wise spatial reorganization with state-space modeling, the proposed GME explicitly exploits the dependency between spatial organization and sequential state propagation. Rather than constructing structural representations from a single fixed spatial arrangement, the four successive stages establish complementary structural dependencies along progressively varied serialization paths, providing a more comprehensive structural representation of heterogeneous breast tumors.

#### 3.2.1 Multi-directional Selective State Space (MSS3D)

The spatial reorganization strategy introduced in GME provides multiple complementary serialization paths for structural representation learning. However, simply reorganizing the spatial arrangement does not establish new structural relationships unless effective dependency modeling is performed under each propagation trajectory. Therefore, an efficient mechanism is required to fully exploit the complementary structural organizations generated by GME and progressively establish long-range structural connectivity.

Recent state-space models (SSMs) have demonstrated remarkable capability in modeling long-range dependencies with linear computational complexity. Unlike self-attention, which explicitly computes pairwise interactions among all tokens, SSMs progressively propagate structural information through recurrent state transitions, making them particularly suitable for volumetric medical image analysis. Motivated by this observation, we propose the Multi-directional Selective State Space (MSS3D) module, which adopts the Mamba operator as the fundamental dependency modeling unit to efficiently establish global structural connectivity under each spatial organization.

Unlike multi-directional Mamba variants that parallelize several scanning directions over the same feature map, MSS3D does not duplicate the Mamba operator along multiple traversal directions. Instead, it relies on a single Mamba operator applied within each spatial organization provided by GME, and complements sequential state propagation with an explicit directional structural regularization along the orthogonal anatomical axes. This design decouples long-range dependency modeling from directional structural continuity: the Mamba operator establishes global connectivity along the reorganized serialization path, while the directional regularization branch compensates for the anisotropy that remains orthogonal to this path, instead of re-scanning the same features along additional directions.

Specifically, the input feature is first normalized and projected through a convolution layer, followed by Mamba-based state-space modeling. A residual connection is further employed to preserve the original structural information while incorporating long-range dependencies, as expressed in Equation 4.

(4) $$X_{m}=X+F_{\text{Mamba}}\left(\mathrm{Conv}\left(\text{LN}\left(X\right)\right)\right),$$

where *F*_Mamba_(·) denotes the selective state-space modeling process for long-range dependency learning.

Although the Mamba operator effectively establishes long-range structural dependencies, its sequential state propagation inherently introduces directional anisotropy, since information is propagated predominantly along the current serialization trajectory. As a result, structural continuity orthogonal to the propagation direction may not be sufficiently modeled. To compensate for this propagation-induced anisotropy, we further introduce a lightweight directional structural regularization branch. Specifically, global average pooling is first adopted to compress the feature representation, as given by Equation 5.

(5) $$X_{p}=\mathrm{Conv}\left(\text{AvgPool}(X_{m})\right),$$

Subsequently, three depth-wise convolutions are independently applied along the depth, height, and width directions to explicitly model directional structural continuity, as formulated in Equation 6.

(6) $$\begin{array}{c} F_{d}={\text{DWC}}_{k\times 1\times 1}(X_{p}),\\ F_{h}={\text{DWC}}_{1\times k\times 1}(X_{p}),\\ F_{w}={\text{DWC}}_{1\times 1\times k}(X_{p}), \end{array}$$

where each branch regularizes structural continuity along one anatomical axis. Compared with isotropic convolution, such directional modeling explicitly preserves complementary structural information distributed across different spatial directions.

The directional structural responses are then concatenated and projected to generate a structural attention map, as defined in Equation 7.

(7) $$A=\sigma \;\left(\mathrm{Conv}\left(\mathrm{Concat}(F_{d},F_{h},F_{w})\right)\right),$$

which is subsequently applied to recalibrate the propagated feature representation.

By jointly integrating Mamba-based state propagation with directional structural regularization, the proposed MSS3D not only establishes long-range structural dependencies, but also compensates for the anisotropy introduced by sequential state propagation. Consequently, structurally complementary information distributed across different anatomical directions can be more effectively preserved, leading to more isotropic representations for heterogeneous breast tumors. Compared with directly scanning the same features along multiple directions and then fusing the results, the combination of GME and MSS3D establishes structural dependencies over genuinely different spatial organizations and then regularizes the remaining directional anisotropy through lightweight axis-wise convolutions, rather than through repeated state propagation over redundant serialization paths.

### 3.3 Gated state-space fusion block

Although the proposed GME block progressively establishes complementary structural dependencies through spatial reorganization and state-space modeling, the resulting representations still exhibit heterogeneous structural characteristics. Specifically, convolutional representations preserve fine-grained local structures and boundary details, whereas state-space representations emphasize long-range structural connectivity. Directly aggregating these complementary representations inevitably weakens their individual advantages and limits the completeness of structural representation.

To address this issue, we propose a Gated State-space Fusion (GSF) block, which performs adaptive structural integration of local and global representations. Instead of directly concatenating or summing features from different branches, GSF dynamically determines their relative contributions according to the structural characteristics of different anatomical regions. The two branches provide complementary structural information, allowing local details and long-range contextual dependencies to be combined within the same feature representation.

As shown in **Figure 3**, the proposed GSF block contains two successive stages: an adaptive gated fusion stage followed by a structure-guided feature interaction stage. Given an input feature $X\in {\mathbb{R}}^{C\times D\times H\times W}$, the convolutional branch first extracts local structural features, as expressed in Equation 8.

**Figure 3:**
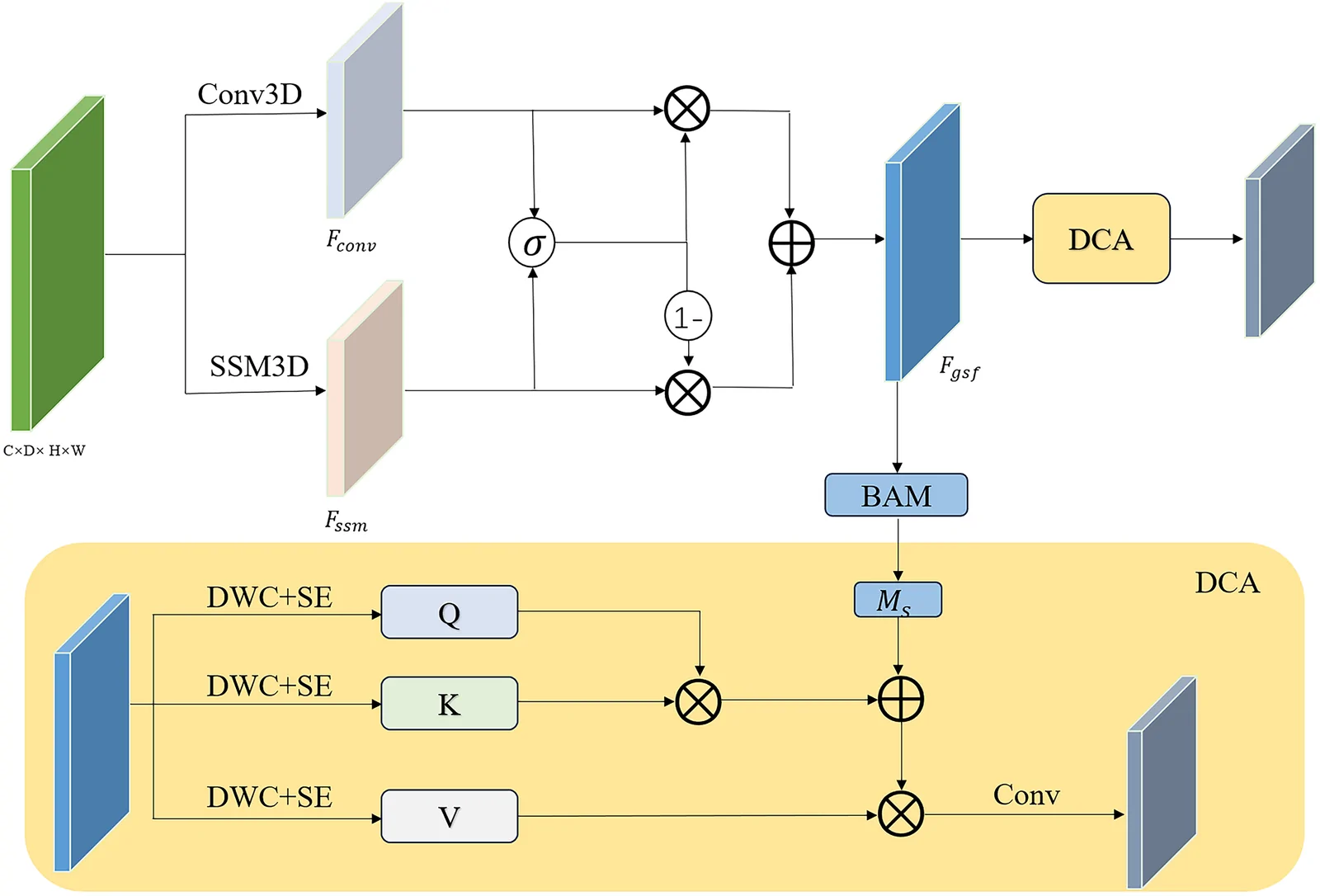
Architecture of the proposed Gated State-space Fusion (GSF) module, consisting of a dual-branch gated fusion mechanism, a Boundary-Aware Modulation (BAM) module, and a Dynamic Channel Attention (DCA) module.

(8) $$F_{conv}=\mathrm{Conv}3\text{D}(X),$$

whereas the state-space branch is used to model long-range structural dependencies, as given by Equation 9.

(9) $$F_{\text{ssm}}=F_{\text{SSM}3\text{D}}(X),$$

where *F*_SSM3D_(·) denotes the state-space modeling operator.

The two branches are not equally important for all spatial regions. To account for this difference, GSF employs a learnable gating mechanism to adjust their contributions according to the input features. The resulting fused representation is written in Equation 10 as

(10) $$F_{\text{gsf}}=G⊙F_{conv}+(1-G)⊙F_{\text{ssm}},$$

where *G* denotes the learnable gating map and ⊙ represents element-wise multiplication.

The obtained fused representation is subsequently forwarded to a structure-guided feature interaction module for further structural refinement. By adaptively integrating complementary structural representations before attention modeling, the proposed GSF block effectively preserves both fine-grained local structures and long-range structural connectivity, providing a more discriminative representation for subsequent boundary-aware feature interaction.

#### 3.3.1 Boundary-aware modulation

Accurate delineation of breast tumors remains challenging because tissue boundaries are usually accompanied by abrupt structural transitions, where structural continuity between adjacent tissues is significantly interrupted. Although the proposed Gated State-space Fusion (GSF) block adaptively integrates complementary local and global structural representations, the subsequent structural interaction process is still primarily driven by feature responses themselves, without explicitly considering structurally transitional regions. Therefore, constructing a structural prior that explicitly characterizes structural variations is essential for enhancing boundary discrimination before structural interaction.

To this end, we propose the Boundary-Aware Modulation (BAM) module, which constructs a boundary-aware structural prior by explicitly modeling structural correlations among spatial locations. Instead of directly relying on feature responses, BAM first interprets the fused feature representation as a learned structural embedding space, where each spatial embedding encodes the local anatomical structure of its corresponding location. Based on this structural embedding space, BAM explicitly estimates the structural consistency between different spatial locations, providing additional structural guidance for subsequent structural interaction.

Given the fused structural representation $F\in {\mathbb{R}}^{C\times D\times H\times W}$, let N=D·H·W denote the number of spatial locations. The feature is first L2-normalized along the channel dimension and reshaped into $F_{\text{norm}}\in {\mathbb{R}}^{C\times N}$. The pairwise structural correlation between spatial locations is then computed in Equation 11 as.

(11) $$Corr=F_{\text{norm}}^{T}F_{\text{norm}}\in {\mathbb{R}}^{N\times N},$$

where *F*_norm_ denotes the normalized structural embedding. Each entry *Corr_ij_* measures the structural consistency between the *i*-th and *j*-th spatial locations by aggregating their channel-wise feature similarity. Thus, BAM performs spatial pairwise correlation rather than channel-wise correlation. The computation requires *O*(*CN*^2^) operations and *O*(*N*^2^) memory for explicitly storing the correlation matrix. In the proposed architecture, BAM is applied only at the bottleneck, after four successive downsampling operations. For an input patch of 64×128×128, the bottleneck spatial resolution is reduced to 4×8×8, resulting in N=256 spatial locations and a 256×256 correlation matrix. This substantially reduces the computational and memory overhead of the pairwise correlation operation and makes it feasible for 3D volumetric feature processing.

In general, regions exhibiting high structural consistency usually belong to homogeneous anatomical tissues, where structural continuity is well preserved and feature responses are highly redundant. Conversely, regions with low structural consistency generally correspond to *structural transition regions*, where tissue boundaries or heterogeneous tumor morphologies introduce significant structural variations. Based on this observation, BAM adopts an inverse correlation modulation strategy to construct the boundary-aware structural prior, as defined in Equation 12.

(12) $$M_{s}=\left(1-\sigma \left(\frac{Corr}{\tau }\right)\right)\lambda +\gamma \sigma \;(1-Corr),$$

where σ(·) denotes the Sigmoid activation function, *τ* is a temperature parameter controlling the smoothness of the response distribution, and λ and γ are the suppression and enhancement coefficients for structurally consistent and structurally transitional regions, respectively.

Unlike conventional correlation-based modulation strategies that only suppress highly correlated responses, the proposed BAM simultaneously suppresses structurally homogeneous regions while enhancing structurally transitional regions. Consequently, the generated modulation map serves as a boundary-aware structural prior, which is subsequently incorporated into the Dynamic Channel Attention (DCA) module to guide structural interaction toward structurally informative regions while suppressing redundant responses from homogeneous anatomical tissues.

#### 3.3.2 Dynamic channel attention

Although the proposed BAM explicitly constructs a boundary-aware structural prior, the structural prior itself cannot directly improve feature representation unless it effectively guides the subsequent structural interaction process. Therefore, it is necessary to incorporate the constructed structural prior into channel dependency modeling, enabling structurally informative regions to participate more actively in feature interaction while suppressing redundant responses from homogeneous anatomical tissues.

To this end, we design a Dynamic Channel Attention (DCA) module to establish structure-guided spatial interaction under the guidance of the boundary-aware structural prior. The term “channel” in the module name refers to the per-location channel recalibration performed by the squeeze-and-excitation (SE) operations in the feature transformation branches *F_Q_*, *F_K_*, and *F_V_*, which recalibrate channel responses before spatial interaction is conducted. Unlike conventional channel attention mechanisms that estimate channel dependencies solely according to feature responses, the proposed DCA further incorporates structural priors into the interaction process, allowing spatial dependency modeling to be adaptively biased toward structurally discriminative regions.

Specifically, three independent transformation branches are first employed to generate the structural query, structural key, and structural value embeddings, as expressed in Equation 13.

(13) $$Q=F_{Q}(F_{\text{gsf}}),\;K=F_{K}(F_{\text{gsf}}),\;V=F_{V}(F_{\text{gsf}}),$$

where FQ(·), FK(·), and FV(·) denote three independent feature transformation branches, each consisting of a depth-wise convolution followed by a squeeze-and-excitation (SE) operation. The depth-wise convolution preserves local structural continuity, while the SE module further recalibrates channel responses to obtain more discriminative structural embeddings.

Let $F_{\text{gsf}}\in {\mathbb{R}}^{C\times D\times H\times W}$ be the input feature to DCA, and let N =D·H·W denote the number of spatial locations. After spatial flattening and transposition, the feature is represented as ${\mathbb{R}}^{N\times C}$. The three transformation branches then produce $Q,K,V\in {\mathbb{R}}^{N\times C}$, where each row corresponds to the structural descriptor of one spatial location and the C channels serve as its feature coordinates.

Based on the generated structural query and key embeddings, the initial structural interaction matrix is computed in Equation 14 as,

(14) $$A=QK^{T}\in {\mathbb{R}}^{N\times N},$$

where *A* characterizes the pairwise interaction strength between spatial locations. Thus, the attention interaction in DCA is spatial-wise rather than channel-wise, with the channel dimension serving as the feature coordinate dimension for computing spatial similarity. This formulation is consistent with the boundary-aware prior $M_{\text{s}}\in {\mathbb{R}}^{N\times N}$ produced by BAM, allowing the two spatial interaction representations to be directly combined in Equation 15.

To explicitly emphasize structurally informative regions, the boundary-aware structural prior generated by BAM is incorporated into the interaction process,

(15) $$A^{\prime }=\text{Softmax}\left(A+M_{\text{s}}\right),$$

where *M*_s_ denotes the boundary-aware structural prior and *A*′ is the refined structural interaction matrix. Guided by the structural prior, feature interaction is encouraged to focus on structurally transitional regions while suppressing redundant interactions originating from structurally homogeneous tissues.

Finally, the refined structural interaction matrix is applied to the structural value embedding, followed by a lightweight convolution layer to aggregate structural information, as given by Equation 16.

(16) $$F_{\text{out}}=\mathrm{Conv}\left(A^{\prime }V\right),$$

where *F*_out_ denotes the output structural representation.

By jointly integrating boundary-aware structural priors with dynamic spatial interaction, the proposed DCA simultaneously models long-range spatial dependencies and emphasizes structurally informative regions. Consequently, complementary features are further refined, leading to more discriminative representations for heterogeneous breast tumors and ambiguous boundary regions. The overall computational complexity of DCA is $O\left(C\cdot N^{2}\right)$ for the two matrix multiplications *QK^T^* and *A*′*V*, while the interaction matrices *A* and *A*′ require $O\left(CN^{2}\right)$ memory. The feature projections *Q*, *K*, and *V* additionally require O(NC) memory, but the overall memory consumption is dominated by the *N*×*N* interaction matrices. Since DCA operates on the same bottleneck feature as BAM, *N* remains small, and the spatial pairwise interaction scales linearly with the channel dimension *C* and quadratically with the already-reduced spatial size *N*.

### 3.4 Adaptive skip recalibration

In encoder–decoder architectures, skip connections are widely adopted to compensate for the loss of spatial information caused by downsampling operations. By directly transmitting low-level detailed features from the encoder to the corresponding decoder stages, skip connections effectively recover spatial resolution and improve the structural completeness of segmentation results. However, conventional skip connections usually employ direct concatenation or addition, which may introduce redundant information and weaken the effectiveness of feature propagation.

To address this issue, we propose an Adaptive Skip Recalibration (ASR) block to enhance the discriminability of encoder features before they are delivered to the decoder. Given the encoder feature at the *l*-th level, denoted as $F_{e}^{l}\in {\mathbb{R}}^{C\times D\times H\times W}$, ASR jointly models spatial and channel dependencies to obtain more informative feature representations.

As illustrated in **Figure 2**, the input feature is first processed by a spatial attention (SA) branch and a channel attention (CA) branch in parallel. The SA branch focuses on salient spatial regions, while the CA branch captures inter-channel dependencies. Their outputs are concatenated along the channel dimension, as expressed in Equation 17.

(17) $$F_{sc}^{l}=\mathrm{Concat}\;\left(SA(F_{e}^{l}),CA(F_{e}^{l})\right).$$

The fused feature is subsequently refined through an additional channel attention module to further emphasize informative channels, as defined in Equation 18.

(18) $$F_{ca}^{l}=CA\left(F_{sc}^{l}\right).$$

Finally, a depthwise convolution (DWC) is employed to enhance local structural representations while maintaining computational efficiency, as formulated in Equation 19.

(19) $$F_{\text{out}}^{l}=\text{DWC}\left(F_{ca}^{l}\right).$$

Through the collaborative modeling of spatial and channel information, ASR adaptively recalibrates encoder features before skip transmission. The spatial attention branch focuses on anatomically relevant regions, while the channel attention branch assigns greater importance to informative feature channels. Channel refinement is then applied to further adjust the feature responses, followed by depthwise convolution to enhance local structural information. These operations help retain discriminative features during propagation and provide clearer representations around ambiguous tumor boundaries.

### 3.5 Loss function

Medical image segmentation is often affected by a substantial imbalance between foreground and background voxels. To provide complementary supervision at both the region and voxel levels, we follow the default loss formulation in nnU-Net and combine Dice Loss with Cross-Entropy (CE) Loss. Dice Loss focuses on the overlap between the predicted and target regions, while CE Loss provides voxel-wise classification supervision.

The Cross-Entropy Loss is defined in Equation 20 as,

(20) $$L_{CE}=-\sum_{n=1}^{N}\sum_{k=1}^{K}t_{n,\;k}\log(s_{n,\;k}),$$

where *N* denotes the number of voxels, *K* is the number of classes, and *t_n_*,*_k_* and *s_n_*,*_k_* represent the ground-truth label and predicted probability of voxel *n* belonging to class *k*, respectively.

The Dice Loss is formulated in Equation 21 as,

(21) $$L_{Dice}=1-\frac{2\sum_{n=1}^{N}\sum_{k=1}^{K}t_{n,\;k}s_{n,\;k}}{\sum_{n=1}^{N}\sum_{k=1}^{K}t_{n,\;k}+\sum_{n=1}^{N}\sum_{k=1}^{K}s_{n,\;k}},$$

where the Dice term measures the overlap between the predicted probability maps and the corresponding target regions.

The overall training objective is given by Equation 22 as,

(22) $$L_{Total}=L_{Dice}+L_{CE}.$$

This combined objective provides complementary supervision: the Dice term encourages accurate region overlap, while the CE term improves voxel-level classification. The formulation is used throughout training without introducing an additional task-specific loss.

### 3.6 Evaluation metrics

Four metrics are used to assess the segmentation results of BAGMNet: Dice, Intersection over Union (IoU), Precision, and Average Surface Distance (ASD). Dice and IoU quantify the overlap between the predicted tumor region and the ground-truth annotation. Precision reflects how many of the predicted tumor voxels are correctly classified, whereas ASD measures the distance between the predicted and reference tumor surfaces. The four metrics therefore cover both region-level agreement and boundary-level accuracy.

The Dice coefficient is defined in Equation 23 as

(23) $$\text{Dice}=\frac{2\left|P\cap G\right|}{\left|P\right|+\left|G\right|},$$

where *P* and *G* denote the predicted region and the ground-truth region, respectively.

The Intersection over Union (IoU) is given by Equation 24 as

(24) $$\text{IoU}=\frac{\left|P\cap G\right|}{\left|P\cup G\right|}.$$

Precision is calculated in Equation 25 as

(25) $$\text{Precision}=\frac{\text{TP}}{\text{TP}+\text{FP}},$$

where TP and FP denote the numbers of true-positive and false-positive voxels, respectively.

The Average Surface Distance (ASD) is defined in Equation 26 as

(26) $$ASD=\frac{1}{\left|S_{P}\right|+\left|S_{G}\right|}\left(\sum_{p\in S\;p}d(p,S_{G})+\sum_{g\in S_{G}}d(g,S_{P})\right),$$

where *S_P_* and *S_G_* denote the boundary point sets of the predicted region and the ground-truth region, respectively, and *d*(·) represents the shortest Euclidean distance from a boundary point to the opposite surface.

For Dice, IoU, and Precision, higher values indicate better segmentation performance. In contrast, a lower ASD value corresponds to more accurate boundary localization and better agreement between the predicted and ground-truth surfaces.

## 4 Results

### 4.1 Datasets

The ISPY1 dataset is derived from the multicenter breast cancer clinical study I-SPY Trial and is a publicly available breast DCE-MRI dataset (31). It contains dynamic contrast-enhanced magnetic resonance imaging scans and corresponding tumor annotations from 161 patients with stage II or stage III breast cancer undergoing neoadjuvant chemotherapy (NACT). Due to substantial inter-patient variations in tumor morphology, size, and enhancement patterns, as well as the heterogeneity of imaging devices and acquisition protocols across different institutions, the dataset poses significant challenges to the robustness of automated segmentation models. In this study, the ISPY1 dataset is employed to train and evaluate the proposed BAGMNet. The dataset is divided into training, validation, and testing sets with a ratio of 8:1:1.

The Yunnan dataset is a publicly available breast DCE-MRI dataset provided by Yunnan Cancer Hospital, consisting of 100 breast cancer cases (32). Each case includes six temporal phases of dynamic contrast-enhanced magnetic resonance imaging (DCE-MRI), comprising one pre-contrast phase and five post-contrast phases, together with breast tumor segmentation annotations manually delineated by experienced radiologists. To maintain consistency with the ISPY1 dataset, the pre-contrast image and the first post-contrast image exhibiting the most prominent enhancement were selected as model inputs, followed by the same preprocessing procedures. Since this dataset originates from a different clinical center with varying imaging devices, acquisition protocols, and tumor appearance characteristics, it is utilized to further evaluate the proposed BAGMNet on an independent multi-center cohort and to assess its robustness across datasets. For experimental settings, the dataset is also partitioned into training, validation, and testing subsets at a ratio of 8:1:1.

### 4.2 Implementation details

The proposed BAGMNet was implemented based on the nnU-Net framework using Python 3.10 and CUDA 11.8. All experiments were conducted on a single NVIDIA RTX 3090 GPU for both training and inference.

All MRI volumes were preprocessed following the nnU-Net pipeline. The images were resampled to an isotropic spacing of 1.0×1.0×1.0 mm^3^ to reduce the effect of spatial resolution differences between cases. Third-order spline interpolation was used for the MRI volumes, whereas nearest-neighbor interpolation was applied to the segmentation masks so that the original label values and boundary locations were preserved.

Voxel intensities were then normalized separately for each volume using Z-score normalization. This step reduces variations in intensity distributions between different scans. After preprocessing, the median volume size was 132×200×200 voxels.

For network training, 3D patches of 64×128×128 voxels were randomly sampled from the preprocessed volumes, with one patch processed per batch. Optimization was performed with stochastic gradient descent (SGD) and Nesterov momentum. The learning rate started at 1×10^−4^, while the weight decay and momentum were set to 3×10^−5^ and 0.99, respectively. Deep supervision was enabled, and training was continued for 200 epochs using the hybrid Dice and Cross-Entropy loss.

For the Mamba modules, *d*_state_ was set to 16 and the expansion factor was set to 2. The same preprocessing procedure and optimization settings were used for all competing methods to keep the comparison consistent.

### 4.3 Analysis of experimental results

#### 4.3.1 Experimental analysis on the ISPY1 dataset

To comprehensively evaluate the effectiveness of the proposed BAGMNet, experiments were conducted on the ISPY1 breast tumor MRI dataset and compared with several representative CNN-based, Transformer-based, and Mamba-based segmentation models, including SegResNet (33), nnUNet (23), UNETR++ (34), SwinUNETR (17), Vmamba (30), Umamba (15), LKM-UNet (35), and HCRT (36). The quantitative comparison results are presented in **Table 1**.

**Table 1:** Quantitative comparison of different methods on the ISPY1 dataset.

Methods	Dice (%) ↑	IoU (%) ↑	ASD (mm) ↓	Precision (%) ↑
SegResNet	65.57	54.51	15.76	65.70
nnUNet	67.54	55.86	10.75	72.97
UNETR++	61.22	49.66	17.27	59.28
SwinUNETR	65.41	54.53	11.40	64.35
Vmamba	62.71	51.34	16.96	63.24
Umamba	63.79	52.11	16.45	63.34
LKM-UNet	64.78	53.98	14.79	69.25
HCRT	69.35	57.65	10.63	74.46
**BAGMNet (Ours)**	**70.34**	**59.48**	**9.79**	**75.32**

The best results are highlighted in bold.

On the ISPY1 dataset, BAGMNet achieves the highest scores for Dice, IoU, and Precision, while also obtaining the lowest ASD among the compared methods, as reported in **Table 1**. The Dice and IoU scores reach 70.34% and 59.48%, respectively, which are 0.99 and 1.83 percentage points higher than those of HCRT. The improvement in both overlap-based metrics and ASD indicates that the proposed method improves the segmentation of the tumor region without sacrificing boundary accuracy. Meanwhile, the ASD is reduced to 9.79 mm and the Precision reaches 75.32%, indicating more accurate boundary localization and fewer false-positive predictions. These results demonstrate that the proposed representation learning strategy effectively captures heterogeneous tumor morphology while preserving anatomically consistent boundaries.

To further assess whether the reported improvement is statistically meaningful, a paired case-level analysis was performed on the main test set. Since the per-case ASD differences deviated from a normal distribution (Shapiro–Wilk test), the Wilcoxon signed-rank test was adopted. The mean paired ASD difference (BAGMNet − HCRT) was −0.84 mm, with a 95% confidence interval of [−2.35, −0.03] mm (*p* = 0.047). The interval lies entirely below zero, indicating that BAGMNet consistently achieves lower ASD than HCRT across cases rather than benefiting from a few outliers. While the Dice improvement is relatively small, the statistically significant ASD reduction confirms that the proposed method provides a reliable advantage in boundary localization, which is the primary design goal of the framework.

To further investigate the effectiveness of the proposed Boundary-Aware Modulation (BAM) mechanism, we visualize its spatial modulation responses on a representative ISPY1 case, as shown in **Figure 4**. The MRI image and the corresponding modulation overlay demonstrate that the learned responses are spatially non-uniform and exhibit strong correspondence with the tumor and its surrounding structural context. The mean-response heatmap further shows that high modulation responses are concentrated around the lesion, indicating that BAM adaptively enhances spatially informative features rather than applying uniform recalibration across the feature map. More importantly, the region-wise analysis reveals that the modulation responses in the boundary and interior regions are consistently higher than those in the exterior background. This suggests that BAM preserves discriminative information within the tumor while maintaining strong responses around its boundary and suppressing irrelevant surrounding features. Such spatially adaptive modulation enables the network to better distinguish tumor boundaries from adjacent tissues, providing complementary structural cues for subsequent feature reconstruction and segmentation.

**Figure 4:**
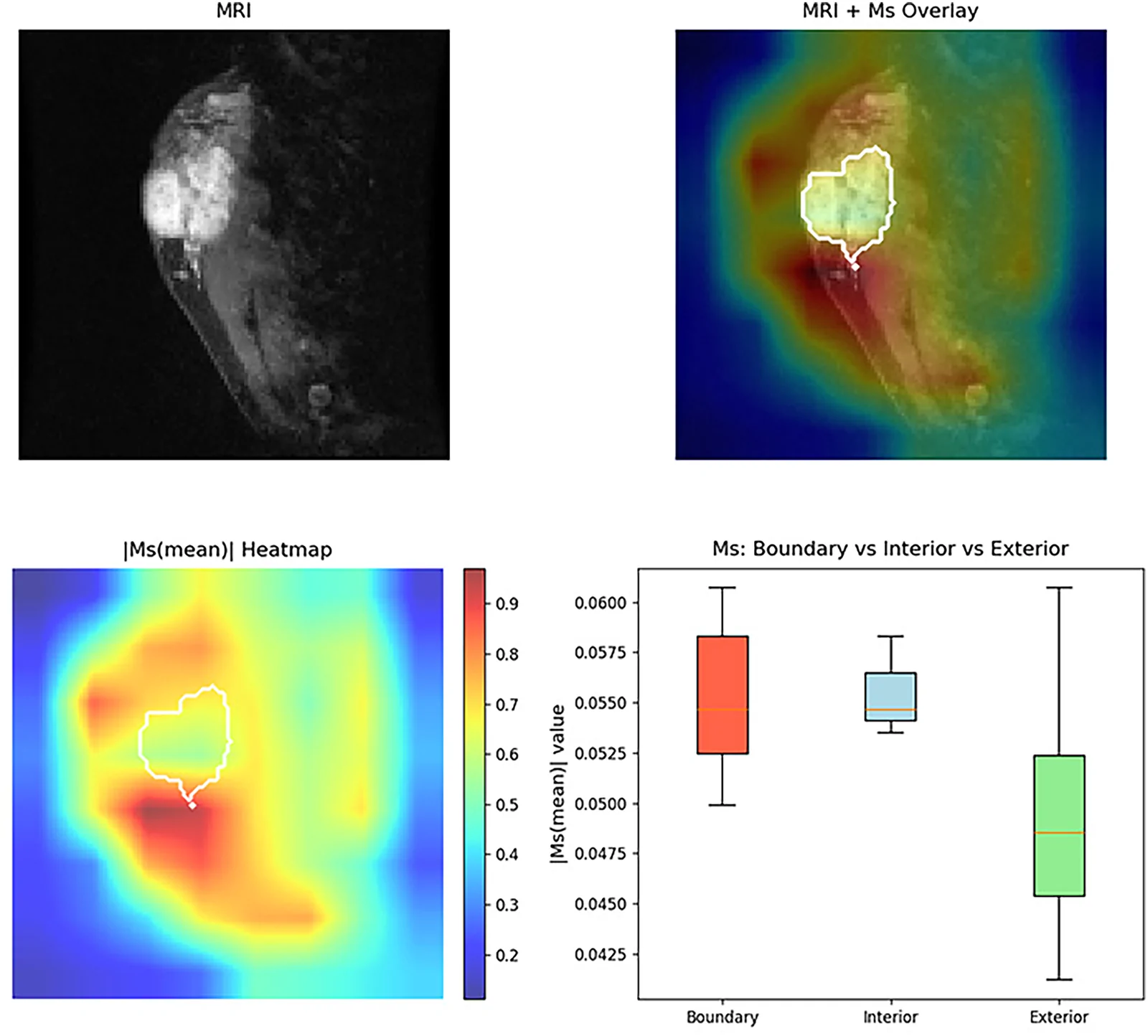
Visualization of the spatially adaptive and boundary-aware responses generated by the proposed BAM module on a representative ISPY1 case.

The superior segmentation performance mainly benefits from the progressive structural representation learning strategy adopted throughout the proposed framework. Specifically, the GME block constructs complementary structural representations through multiple geometric transformations, enabling the network to perceive morphological variations under different spatial arrangements. Building upon these representations, the MSS3D module enhances long-range structural connectivity via multi-directional state-space modeling, thereby alleviating the anisotropy introduced by sequential state propagation. Furthermore, the GSF module adaptively integrates complementary local structural details and global contextual representations while introducing boundary-aware structural priors to guide feature interaction. Finally, the ASR block recalibrates encoder features before skip transmission, preserving structurally informative representations for accurate anatomical structure reconstruction during decoding. The collaborative optimization of these modules enables the proposed framework to achieve superior performance in both region segmentation and boundary delineation.

The qualitative visualization results shown in **Figure 5** further demonstrate the superiority of the proposed method. Traditional CNN-based approaches tend to produce boundary discontinuities and contour deviations in thin and elongated tumor regions due to their limited receptive fields. Although Transformer- and Mamba-based methods enhance global information interaction, they still suffer from boundary over-smoothing and detail loss in complex boundary areas. In particular, LKM-UNet exhibits obvious boundary over-expansion, while Vmamba and Umamba show partial structural omission in thin boundary regions. By contrast, BAGMNet produces segmentation results that are substantially more consistent with the ground truth. In particular, the proposed method successfully preserves the thin protruding structure extending from the left side of the lesion, whereas several competing methods either over-smooth this region or fail to recover it completely. Meanwhile, obvious false-positive responses observed in methods such as LKM-UNet are effectively suppressed by the proposed framework. These observations indicate that the proposed structural representation learning strategy is capable of preserving fine anatomical structures while maintaining boundary continuity and reducing over-segmentation.

**Figure 5:**
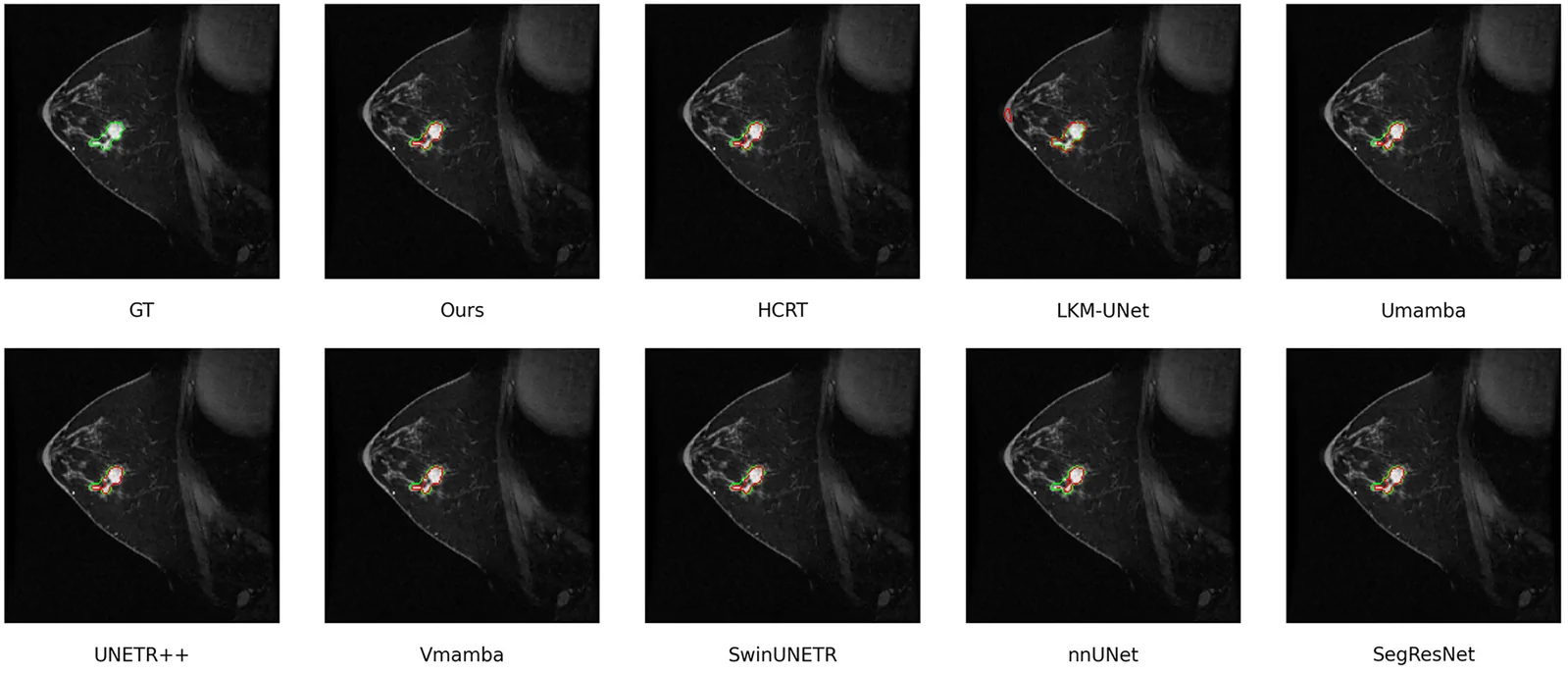
Qualitative comparison of different segmentation methods on representative breast tumor MRI cases from the ISPY1 dataset.

Moreover, these qualitative observations are highly consistent with the superior ASD and Precision reported in **Table 1**, further demonstrating the effectiveness of the proposed framework for accurate boundary delineation in heterogeneous breast tumor segmentation.

#### 4.3.2 Experimental analysis on the Yunnan dataset

To further evaluate the proposed BAGMNet on an independent dataset from a different clinical center, experiments were conducted on the Yunnan dataset and compared with several representative CNN-based, Transformer-based, and Mamba-based segmentation methods. Considering that breast tumor segmentation requires not only accurate region prediction but also reliable boundary delineation, both overlap-based metrics (Dice and IoU) and boundary-aware metrics (ASD and Precision) were adopted for comprehensive evaluation. The quantitative comparison is summarized in **Table 2**.

**Table 2:** Quantitative comparison of different methods on the Yunnan dataset.

Methods	Dice (%) ↑	IoU (%) ↑	ASD (mm) ↓	Precision (%) ↑
SegResNet	76.53	65.56	10.48	73.96
nnUNet	74.19	63.64	12.87	74.34
UNETR++	71.95	60.48	12.83	77.48
SwinUNETR	70.73	59.46	13.74	70.62
Vmamba	73.66	62.95	15.47	74.46
Umamba	74.80	63.79	14.16	75.65
LKM-UNet	68.75	57.98	15.44	76.74
HCRT	**79.54**	**69.65**	9.25	80.34
**BAGMNet (Ours)**	79.14	69.48	**8.96**	**82.91**

The best results are highlighted in bold.

As shown in **Table 2**, BAGMNet achieves the best performance in both ASD and Precision, obtaining an ASD of 8.96 mm and a Precision of 82.91%. Compared with HCRT, BAGMNet achieves a 0.29 mm reduction in ASD and a 2.57 percentage-point improvement in Precision, suggesting more accurate boundary localization and better control of false-positive predictions. BAGMNet obtains Dice and IoU scores of 79.14% and 69.48%, respectively, differing from HCRT by only 0.40 and 0.17 percentage points. Although the highest Dice score is achieved by HCRT, BAGMNet performs better in ASD and Precision. The lower ASD suggests more accurate boundary localization, while the higher Precision indicates fewer false-positive predictions. Thus, the advantage of BAGMNet is reflected not only in region overlap but also in the quality of the predicted tumor boundaries. The boundary-aware structural modeling strategy encourages conservative predictions around ambiguous tumor boundaries, effectively suppressing false-positive responses while preserving anatomically meaningful structures. As a result, the proposed method produces more reliable structural representations with only a marginal reduction in overlap-based metrics.

The superior boundary localization can be mainly attributed to the progressive structural representation learning strategy adopted throughout the proposed framework. Specifically, the GME block performs spatial reorganization through multiple geometric transformations to construct complementary structural representations. Subsequently, the MSS3D module enhances long-range structural connectivity via multi-directional state propagation, enabling the network to perceive heterogeneous tumor structures from different spatial orientations. The GSF block further integrates complementary local and global structural representations and introduces boundary-aware structural priors to guide structure-aware feature interaction. Finally, the ASR block recalibrates encoder features before cross-level transmission, preserving structurally informative representations for accurate anatomical structure reconstruction during decoding.

Overall, the experimental results on the Yunnan dataset demonstrate that BAGMNet achieves an effective trade-off between regional overlap and boundary delineation quality on an independent multi-center cohort. Although its Dice score is slightly lower than that of HCRT, the proposed framework consistently produces more reliable structural representations, leading to superior boundary localization accuracy and fewer false-positive predictions. These results suggest that BAGMNet places greater emphasis on structurally discriminative features rather than region overlap alone, which helps preserve tumor boundaries in heterogeneous breast lesions.

### 4.4 Robustness analysis via repeated random splits

Although a fixed training–validation–testing split provides a consistent setting for comparison with existing methods, the limited number of cases in medical imaging datasets can make the reported performance sensitive to the particular data partition. To examine the stability of BAGMNet under different data splits, we further performed a repeated random split experiment on both the ISPY1 and Yunnan datasets.

For each dataset, the cases were randomly shuffled and divided into training, validation, and testing sets at a ratio of 8:1:1 using five different random seeds. The model was independently trained and evaluated for each split under the same settings as those used in the main experiments. The mean performance and standard deviation over the five runs are reported in **Table 3**.

**Table 3:** Robustness analysis results under five repeated random splits.

Dataset	Dice (%) ↑	IoU (%) ↑	ASD (mm) ↓	Precision (%) ↑
ISPY1	71.36 ± 2.30	59.69 ± 1.89	9.66 ± 2.16	74.86 ± 2.89
Yunnan	79.23 ± 1.25	69.34 ± 1.44	8.03 ± 1.70	81.56 ± 2.36

As shown in **Table 3**, BAGMNet maintains relatively stable performance across the different random splits. On the ISPY1 dataset, the model achieves a mean Dice of 71.36% ± 2.30% and an IoU of 59.69% ± 1.89%, with an ASD of 9.66 ± 2.16. On the Yunnan dataset, the corresponding Dice and IoU scores are 79.23% ± 1.25% and 69.34% ± 1.44%, respectively, while the ASD is 8.03 ± 1.70. The relatively small variations across the five splits suggest that the segmentation performance is not strongly dependent on a particular data partition, providing additional evidence of the stability of the proposed method.

### 4.5 Ablation study

#### 4.5.1 Component ablation study

The contribution of GME, GSF, and ASR was evaluated on the ISPY1 dataset using U-Mamba as the baseline. The three modules were added sequentially to the baseline network, with the corresponding results presented in **Table 4**. GME introduces geometric views for structural representation, GSF handles the interaction between local and long-range features, and ASR recalibrates features passed through the skip connections.

**Table 4:** Component ablation study on the ISPY1 dataset.

Method	GME	GSF	ASR	Dice (%) ↑	ASD (mm) ↓
Baseline (U-Mamba)	×	×	×	63.79	16.45
+ GME	✓	×	×	67.16	12.65
+ GSF	×	✓	×	66.03	13.79
+ GME + GSF	✓	✓	×	69.17	10.23
**+ GME + GSF + ASR (Ours)**	✓	✓	✓	**70.34**	**9.79**

Bold values indicate the best performance among the compared methods.

Starting from the baseline, the Dice score increases from 63.79% to 70.34% after all three modules are included. At the same time, ASD drops from 16.45 mm to 9.79 mm. The changes in these two metrics suggest that the added components improve both region overlap and boundary localization.

The first noticeable gain comes from GME. With this module, Dice increases by 3.37 percentage points and ASD decreases by 3.80 mm relative to U-Mamba. GME exposes the encoder to several geometric arrangements of the same feature representation, providing additional structural cues for subsequent state-space modeling. This appears to be particularly useful for tumors with irregular morphology and poorly defined boundaries.

Introducing the GSF module alone also leads to noticeable performance improvement. By performing structure-aware feature interaction between local structural details and global contextual representations, the proposed module enhances the discriminability of high-level semantic features and facilitates more effective structural information aggregation.

When both GME and GSF are incorporated simultaneously, segmentation performance is further improved, with the Dice score increasing to 69.17% and the ASD decreasing to 10.23 mm. Compared with using either module individually, their combination achieves substantially greater performance gains, demonstrating that multi-view structural representation learning and structure-aware feature interaction complement each other in modeling heterogeneous breast tumor structures.

Finally, the ASR module is introduced to further preserve structurally informative representations during encoder–decoder interaction. By adaptively recalibrating encoder features before skip transmission, ASR effectively suppresses redundant background responses while enhancing boundary-related structural information. Consequently, the complete framework achieves the best overall performance, obtaining a Dice score of 70.34% and an ASD of 9.79 mm.

Overall, the progressive gains observed in the ablation experiments support the effectiveness of the proposed structural representation framework. The three modules play different roles within the network. GME enriches the spatial views available to the encoder, while GSF and ASR refine feature interaction and skip information during decoding. Their successive addition leads to consistent gains in both segmentation accuracy and boundary quality.

#### 4.5.2 GME block ablation

The effect of the number of geometric views in GME was investigated on the ISPY1 dataset. Four configurations were compared, starting from the identity view and progressively adding depth-wise flipping, depth–height transposition, and the final SpatialFlip transformation across successive encoder stages. The results are given in **Table 5**.

**Table 5:** Ablation study of different structural-view configurations in the proposed GME block.

Configuration	Structural Views	Dice (%) ↑	ASD (mm) ↓
Single-view	Original	66.19	14.64
Two-view	Original + Flip	67.84	12.49
Three-view	Original + Flip + Transpose	69.37	10.48
**Four-view (Ours)**	**Original + Flip + Transpose + SpatialFlip**	**70.34**	**9.79**

Bold values indicate the best performance among the compared methods.

The single-view configuration obtains a Dice score of 66.19% and an ASD of 14.64 mm. Adding the Flip transformation raises Dice to 67.84% and reduces ASD to 12.49 mm. A further gain is observed after introducing the Transpose transformation, with Dice reaching 69.37% and ASD decreasing to 10.48 mm. Exchanging the depth and height dimensions provides the model with another spatial arrangement of the feature representation, which can expose structural relationships that are less apparent in the original orientation.

The complete four-view configuration achieves the highest scores, reaching 70.34% Dice and 9.79 mm ASD. Relative to the single-view setting, this corresponds to a gain of 4.15 percentage points in Dice and a reduction of 4.85 mm in ASD. The changes in both metrics are consistent across the progressive configurations, supporting the use of multiple geometric views for capturing the varied spatial structures of breast tumors.

We note that the geometric transformations used in GME are parameter-free reshape and indexing operations that introduce no additional FLOPs. Since each encoding stage employs a single MSS3D module regardless of the assigned transformation, all four configurations share the same number of parameters (7.73 M) and computational cost (80.29 G FLOPs on a 64×128×128 input patch). The observed improvement from 66.19% to 70.34% Dice and from 14.64 mm to 9.79 mm ASD is therefore entirely attributable to the complementary structural dependencies established under different spatial organizations, rather than to any increase in model capacity or computation.

Overall, the ablation results support the use of multi-view geometric modeling in GME. Combining complementary spatial views provides richer structural cues for the subsequent state-space modeling, which is particularly useful for representing the heterogeneous morphology and ambiguous boundaries of breast tumors.

#### 4.5.3 MSS3D ablation

To examine the respective contributions of state-space modeling and multi-directional anisotropy compensation in the proposed Multi-directional Selective State Space (MSS3D) module, three configurations were compared. The first replaces the state-space branch with a conventional convolutional branch (Conv substitute). The second retains only the Mamba branch for long-range dependency modeling (Mamba only). The final configuration corresponds to the complete MSS3D module, which combines Mamba-based state propagation with the proposed multi-directional anisotropy compensation. The results are summarized in **Table 6**.

**Table 6:** Ablation study of the proposed MSS3D module.

Method	Dice (%) ↑	ASD (mm) ↓
Conv substitute	67.68	13.66
Mamba only	69.39	11.95
**Ours**	**70.34**	**9.79**

Bold values indicate the best performance among the compared methods.

With the Conv substitute configuration, the model obtains a Dice score of 67.68% and an ASD of 13.66 mm. Although the convolutional branch is effective at extracting local spatial patterns, its limited receptive field makes long-range interaction across volumetric features more difficult, resulting in weaker segmentation performance.

Adding the Mamba branch increases Dice to 69.39% and reduces ASD to 11.95 mm. The improvement in both metrics suggests that long-range state-space modeling provides additional contextual information and allows features to interact over larger spatial distances, which is useful for representing complex tumor structures.

Finally, incorporating the proposed multi-directional anisotropy compensation mechanism further improves the Dice score to 70.34% and reduces ASD to 9.79 mm. Compared with the Mamba-only configuration, Dice is further improved by 0.95 percentage points, while ASD is reduced by 2.16 mm. These results indicate that although Mamba effectively models long-range dependencies, its sequential scanning process inevitably introduces anisotropy. By explicitly enhancing feature responses along multiple spatial directions, the proposed MSS3D module effectively compensates for this anisotropy and produces more consistent structural representations for accurate boundary delineation.

The ablation results highlight the different roles of the two components. Mamba mainly contributes to long-range state propagation, while the multi-directional design introduces additional spatial directions for feature interaction. Using both components gives MSS3D a broader contextual view without relying on a single scanning direction, which is consistent with the improvement observed in the segmentation results.

#### 4.5.4 GSF block ablation

The individual components of the GSF block were evaluated through a progressive ablation study on the ISPY1 dataset. Starting from the baseline configuration, the dual-branch architecture, adaptive gating, Dynamic Channel Attention (DCA), and Boundary-Aware Modulation (BAM) were introduced successively. The results are reported in **Table 7**.

**Table 7:** Ablation study of the proposed GSF block.

Method	Dice (%) ↑	ASD (mm) ↓
Baseline	68.12	12.06
+ Dual-branch	68.61	11.82
+ Adaptive Gate	68.92	11.65
+ DCA	69.12	10.72
**+ BAM (Ours)**	**70.34**	**9.79**

Bold values indicate the best performance among the compared methods.

The baseline records a Dice score of 68.12% with an ASD of 12.06 mm. After introducing the dual-branch design, these values change to 68.61% and 11.82 mm, respectively. The gain indicates that the convolutional and state-space branches capture different types of information: convolution preserves local structural patterns, whereas the state-space branch provides longer-range contextual information.

The next configuration adds the gating mechanism to the two-branch structure. Dice reaches 68.92% and ASD falls to 11.65 mm. Rather than treating the two branches equally, the gate adjusts their relative contributions according to the input features, making the resulting fusion more selective.

DCA produces a further change in the boundary-related metric. With this component, Dice increases to 69.12%, while ASD drops to 10.72 mm. The larger reduction in ASD compared with the Dice gain suggests that channel-wise recalibration mainly benefits the preservation of structurally informative features.

The largest improvement appears after introducing BAM. The complete configuration reaches 70.34% Dice and 9.79 mm ASD, corresponding to gains of 1.22 percentage points and 0.93 mm over the configuration without BAM. This result indicates that the boundary-aware modulation provides additional structural cues that are useful for refining ambiguous tumor regions. The larger improvement in ASD suggests that BAM has a more direct effect on boundary-related representation. By estimating structural consistency in the feature space and reducing responses from homogeneous regions, BAM provides additional guidance for the subsequent attention operation, allowing it to assign greater importance to ambiguous boundary regions.

Taken together, the ablation results show a gradual improvement as the different components are incorporated. The dual-branch design establishes complementary local and long-range feature modeling, while gating and channel attention further refine feature interaction. BAM provides an additional structural constraint around tumor boundaries, leading to the final improvement in both region overlap and boundary localization.

### 4.6 Ablation study of boundary modeling strategies

To validate the effectiveness of the proposed Boundary-Aware Modulation (BAM) module, we conducted an ablation study on different boundary modeling strategies. While keeping the network architecture, training strategy, and other components unchanged, we employed the Sobel operator (37), Boundary-Aware Gradient Operator (BAGO) (38), and the proposed BAM for boundary modeling. The results are presented in **Table 8**.

**Table 8:** Ablation study of different boundary modeling strategies.

Boundary modeling	Dice (%) ↑	ASD (mm) ↓
Sobel	69.32	12.47
BAGO	69.73	10.61
**BAM (Ours)**	**70.34**	**9.79**

Bold values indicate the best performance among the compared methods.

As shown in **Table 8**, the Sobel, BAGO, and BAM variants achieve Dice scores of 69.32%, 69.73%, and 70.34%, with corresponding ASD values of 12.47, 10.61, and 9.79, respectively. Compared with the fixed-gradient Sobel operator, BAGO achieves improved performance through learnable boundary gradient modeling, indicating that learnable boundary features can better characterize ambiguous tumor boundaries in breast MRI. Furthermore, BAM achieves the best performance on both Dice and ASD, improving the Dice score by 0.61 percentage points and reducing ASD by 0.82 compared with BAGO. These results demonstrate that BAM can adaptively modulate spatial features according to the input context, thereby enhancing discriminative representations around tumor regions and boundaries and improving the segmentation of complex tumor boundaries.

## 5 Conclusion

This paper proposed a Boundary-Aware Geometric Mamba Network (BAGMNet) for accurate and robust breast tumor segmentation in 3D MRI. Different from existing CNN-, Transformer-, and Mamba-based methods that are limited by insufficient structural perception and ambiguous boundary modeling, BAGMNet establishes a structural representation learning framework to enhance three-dimensional tumor understanding.

Specifically, BAGMNet introduces multi-view geometric representation learning to capture diverse tumor morphology, employs multi-directional state-space modeling to alleviate the anisotropy caused by conventional Mamba scanning, and adopts structure-guided feature refinement to preserve discriminative boundary information during feature fusion and decoding. These designs enable more comprehensive structural perception and accurate tumor delineation.

Extensive experiments on the multicenter ISPY1 dataset and an independent Yunnan breast MRI dataset demonstrate the effectiveness and multi-dataset robustness of BAGMNet. Compared with representative CNN-, Transformer-, and Mamba-based methods, BAGMNet achieves competitive segmentation performance with improved boundary localization and structural consistency. Furthermore, repeated random-split experiments and ablation studies verify the stability of the proposed framework and the complementary contributions of each component.

Despite its promising performance, the proposed framework still has some limitations. The boundary-aware structural modeling introduces additional computational costs, which may affect scalability for high-resolution volumetric images. In addition, further validation on larger multicenter cohorts and other imaging modalities is required to evaluate its clinical applicability.

Future work will investigate more efficient structural interaction mechanisms and the integration of multi-modal imaging and clinical prior information. The applicability of BAGMNet to other volumetric medical image segmentation tasks will also be explored.

## Data Availability

Publicly available datasets were analyzed in this study. The ISPY1 dataset is publicly available from The Cancer Imaging Archive (TCIA): https://www.cancerimagingarchive.net/collection/ispy1/. The Yunnan breast MRI dataset is described in DOI: 10.1016/j.patter.2023.100826.
